# Technical advance in silico and in vitro development of a new bipolar radiofrequency ablation device for renal denervation

**DOI:** 10.1186/s12872-021-02305-x

**Published:** 2021-10-16

**Authors:** Noel Pérez, Karl Muffly, Stephen E. Saddow

**Affiliations:** 1 Department of Electrical Engineering and Department of Medical Engineering, University of South Florida, Tampa, FL USA; 2 Departments of Pathology and Cell Biology and Surgery, University of South Florida, Tampa, FL USA; 3 Research and Development, Oscor Inc, Palm Harbor, FL USA

**Keywords:** Basket catheter, High blood pressure, Hypertension, Radiofrequency ablation, Renal denervation, Resistant hypertension

## Abstract

**Background:**

Renal denervation with radiofrequency ablation has become an accepted treatment for drug-resistant hypertension. However, there is a continuing need to develop new catheters for high-accuracy, targeted ablation. We therefore developed a radiofrequency bipolar electrode for controlled, targeted ablation through Joule heating induction between 60 and 100 °C. The bipolar design can easily be assembled into a basket catheter for deployment inside the renal artery.

**Methods:**

Finite element modeling was used to determine the optimum catheter design to deliver a minimum ablation zone of 4 mm (W) × 10 mm (L) × 4 mm (H) within 60 s with a 500 kHz, 60 Vp-p signal, and 3 W maximum. The in silico model was validated with in vitro experiments using a thermochromic phantom tissue prepared with polyacrylamide gel and a thermochromic ink additive that permanently changes from pink to magenta when heated over 60 °C.

**Results:**

The in vitro ablation zone closely matched the size and shape of the simulated area. The new electrode design directs the current density towards the artery walls and tissue, reducing unwanted blood temperature increases by focusing energy on the ablation zone. In contrast, the basket catheter design does not block renal flow during renal denervation.

**Conclusions:**

This computational model of radiofrequency ablation can be used to estimate renal artery ablation zones for highly targeted renal denervation in patients with resistant hypertension. Furthermore, this innovative catheter has short ablation times and is one of the lowest power requirements of existing designs to perform the ablation.

## Background

Hypertension increases the risk of heart disease, stroke, and death. A 12–13 mmHg reduction in systolic blood pressure reduces the risk of cardiovascular disease by at least 21% [1]. In 2020, the American Heart Association (AHA) reported that 31.9% of Americans have hypertension, resulting in a healthcare expenditure of $55.9 billion [2]. AHA/American College of Cardiology guidelines define resistant hypertension (RH) as hypertension resistant to three or more medications, resulting in higher blood pressure than a ≥ 130/80 mmHg target or a blood pressure < 130/80 mmHg in a patient taking ≥ 4 antihypertensive drugs. RH affects 12–15% of patients treated for hypertension and remains a clinical management challenge [3].

Hypertension is, at least in part, caused by chronic activation of the sympathetic nervous system. As early as 1889, experiments with dogs showed that renal or splanchnic nerves stimulation caused changes in blood pressure [4]. In 1925, Adson performed surgical sympathectomy (disconnection of the sympathetic nerves at the trunk) to treat hypertension [5]. For the subsequent two decades, surgical sympathectomy (thoracolumbar splanchnicectomy) became the procedure of choice for patients with HT who did not respond to diet or the then limited available pharmacological therapy [6]. Indeed, between 1938 and 1947, approximately 2400 patients with hypertension were treated by surgical sympathectomy [7]. Although blood pressure reductions after the operation were often significant, patients were seriously affected by intolerable post-operative postural hypotension [8]. When the first effective antihypertensive drugs (diuretics) became available in the mid-1950s, pharmacological therapy became the standard of care for patients with hypertension [9].

Since stimulation of the renal nerves raises blood pressure through vasoconstriction and volume and sodium retention [10–12], ablating renal nerve activity (renal denervation; RD) effectively lowers blood pressure [13]. The highest density of renal sympathetic nerves is within 12–15 mm of the abdominal aorta and within 4 mm radially from the renal artery inner lumen [14]. RD's target is to disconnect the efferent and afferent renal sympathetic nerves between the kidneys and the central nervous system to reduce blood pressure. Indeed, the first bilateral RDs for hypertension were performed in 1934 and 1935, establishing the procedure's safety with no adverse effect on renal function [15, 16].

Multiple RD methods are incorporated in devices using radiofrequency, chemicals, or ultrasound [13]. However, an RD human trial in the USA, SYMPLICITY HTN-3, did not meet the primary efficacy endpoint [17]. Still, subsequent trials using next-generation multielectrode radiofrequency (RF) and ultrasound renal nerve ablation systems have shown consistent blood pressure-reducing effects [11, 12]. The SYMPLICITY HTN-3 trial resulted in the redesign of the ablation catheter from a single point unipolar ablation electrode to a four-quadrant unipolar device.

To date, no RD device has been cleared by the Food and Drug Administration (FDA) in the USA except for investigational purposes [18]. Here we employed a computerized (in silico*)* finite element model (FEM) to optimize an innovative bipolar RF ablation device. The model was validated with thermochromic phantom (TCP) tissue by comparing ablation zone geometries. Our new catheter design does not block renal blood flow, does not require cooling, and has low power consumption due to the bipolar electrode design.

## Methods

### Computational modeling

The finite-element method (FEM) has previously been used to model RF tumor or cardiac ablation [19–21]. Here we also used FEM to solve electromagnetic equations coupled with bioheat equations for bipolar electrodes in contact with tissue design to deliver a minimum ablation zone of 4 mm (W) × 10 mm (L) × 4 mm (H) within 60 s with a 500 kHz, 60 Vp-p signal and 3 W maximum.

COMSOL version 5.6 (COMSOL, Stockholm, Sweden) was used to generate geometric models, assign material properties and boundary conditions, define infinite element domains, generate meshes, and perform the coupled electromagnetic heating calculations. A coupled analysis was required since the electrical properties of tissue change with temperature and, therefore, the electric field profile must be recalculated at each time step. All analyses were performed on a PC equipped with an Intel® Core™ i7-9700 CPU @ 3 GHz, 64 GB of memory, and Windows 10 Home OS.

#### Model geometry

**Fig. 1:**
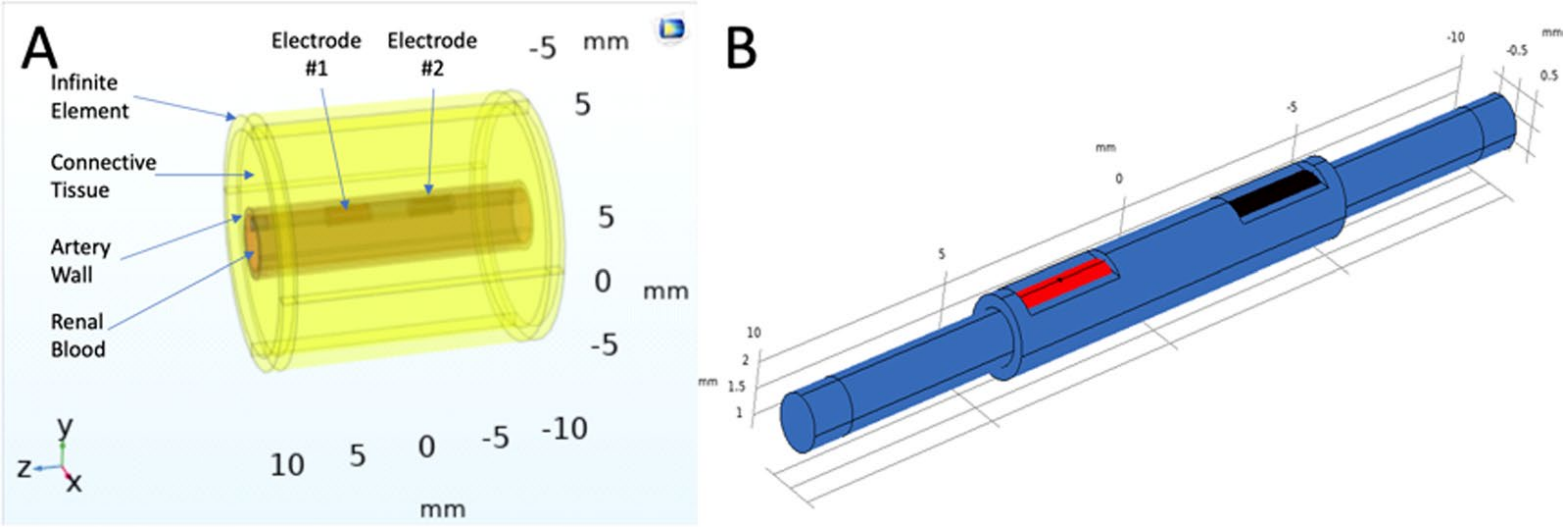
**A** 3D model geometry for FEM analysis of the bipolar electrodes in contact with phantom tissue to mimic placement inside the renal artery. Electrode dimensions: diameter 0.61 mm, length 3 mm with a 1.5 mm separation. **B** Innovative bipolar electrode design (patent pending) focuses current density towards the artery wall and surrounding tissue—electrodes (red and black) dimensions as per (**A**)

Multiple software packages are commercially available to perform FEM for solving partial differential equations. We used the readily available COMSOL software to develop a 3D FEM model geometry of bipolar cylindrical electrodes inside a renal artery surrounded by connective tissue (Fig. 1A). The renal artery internal diameter was 4.1 mm [14], the wall (tunica intima and media) thickness was 0.5 mm [22], and the surrounding connective tissue was modeled to a radius of 10 mm. An infinite element domain was defined around the geometric model for approximating an infinitely large domain (Fig. 1A). The electrode dimensions were 0.61 mm outside diameter, 0.1 mm thickness, and 3 mm long assembled over a Pellethane® tube. The electrodes were designed to be in contact with the renal artery's inner wall through an exposed window of 1 mm × 2.5 mm, as shown in Fig. 1B (patent pending).

#### Electrical and thermal properties

Table 1 shows the material properties at 500 kHz used in the computational model to describe the blood, artery wall, connective tissue, thermoplastic catheter material (Pebax®), and electrode components.

**Table 1:** Electrical and thermal properties at 500 kHz for components in the FEM model

Component	Relative permittivity (ε_r_)	Electrical conductivity σ (S/m)	Density (kg/m^3^)	Heat capacity at constant pressure J/(kg °C)	Thermal conductivity W/(m °C)
Blood^a^	4190	0.748	1050	3617	0.52
Artery wall^a^	312	0.324	1102	3306	0.46
Connective tissue^a^	201	0.391	1027	2372	0.39
Pebax® 7233^b^	4	1.25E−12	1010	1700	0.26
Electrodes (PtIr 90/10)^c,d^	1	1.00E+09	7950	502	14

^a^Dielectric properties» IT’IS Foundation. https://itis.swiss/virtual-population/tissue-properties/database/dielectric-properties/

^b^https://www.materialdatacenter.com/ms/en/tradenames/Pebax/ARKEMA/Pebax%C2%AE+7233+SP+01/f21dd478/264

^c^https://www.engineersedge.com/properties_of_metals.htm

^d^https://www.azom.com/properties.aspx?ArticleID=965

Temperature changes result in electrical conductivity changes in biological tissues (artery walls and connective tissue) [23] and, during RF ablation, the temperature of tissues increases. The changes to the electrical conductivity in tissues included in the model were described using:

1 $$\sigma \left( T \right) = \mathop \sigma \limits_{^\circ } \left[ {1 + k_{1} \Delta T} \right]$$

where $\mathop \sigma \limits_{^\circ }$ is the initial electrical conductivity at the reference temperature, $k_{1}$ is the temperature coefficient, and $\Delta T$[°C] is the temperature difference from the initial reference temperature. We set $k_{1} = 2.05\%$/°C, and the linear electrical conductivity in the model was determined by

2 $$\sigma \left( T \right) = 0.324\left[ {1 + 0.0205\Delta T} \right]$$

For the artery wall and connective tissue, the equation was

3 $$\sigma \left( T \right) = 0.391\left[ {1 + 0.0205\Delta T} \right]$$

Both equations are bounded within the experimental data of 30–80 °C [24].

### Multi-physics computation

#### Electric field

When electric fields are applied to tissue, the temperature in the zone between the bipolar electrodes increases due to Joule heating through tissue conduction and dielectric loss. For the tissues in the model at 500 kHz, dielectric losses are negligible, so only the resistive loss (i.e., Joule heating) was considered in this simulation [25]. The electric field **E** (V/m) profile was calculated from the negative gradient of the voltage using the equation

4 $${\mathbf{E}} = - \nabla {\mathbf{V}}$$

where ∇ is the *del* operator that turns the scalar voltage **V** (V) to a vector field. Then, current density **J** (A/m^2^) was computed from

5 $${\varvec{J}} = \frac{{\mathbf{E}}}{\rho e}$$

where $\rho e$ is the electrical resistivity of the material (Ω m). Then, the average power dissipated in the tissue between the electrodes was determined by

6 $$P_{{{\text{av}}}} = \frac{1}{2}\Re \left( {{\varvec{J}}{*} \cdot {\varvec{E}}} \right)$$

where $\Re$ is the real part from the vector dot product of conjugate ${\varvec{J}}$ and ${\varvec{E}}$.

#### Bioheat equation

Penne’s bioheat equation describes the temperature distribution increase surrounding the bipolar electrodes during the Joule heating process and incorporates the convective heat transfer from blood perfusion within the tissue

7 $$\begin{aligned}\rho C_{{\text{p}}} \frac{\partial T}{{\partial t}} - \nabla \cdot k\nabla T &= \frac{1}{2}\Re \left( {{\varvec{J}}* \cdot {\varvec{E}}} \right)\\ &\quad+ \rho_{{\text{b}}} C_{{\text{p,b}}}\upomega _{{\text{b}}} \left( {T_{{\text{b}}} - T} \right) + Q_{{{\text{met}}}}\end{aligned}$$

where $\rho$ is the tissue density (kg/m^3^), *C*_*p*_ is the tissue-specific heat (J/kg K), and *k* is the tissue thermal conductivity (W/m K) of the components in the model. T_b_ is the blood temperature (assumed to be 37 °C), $\rho_{{\text{b}}}$ is blood density (kg/m^3^), *C*_*p,b*_ the specific heat of the blood (J/kg K), and ω_*b*_ is the blood perfusion (1/s). *Q*_*met*_ is the energy generated by metabolic processes (W/m^3^) and is negligible for the renal artery model. The blood temperature in large surrounding vessels has minimal impact on the thermal field between the bipolar electrodes due to their distance from the renal artery and was therefore not included in the model.

#### Boundary conditions

Artery walls and surrounding tissue with Dirichlet boundary conditions: temp = normal body temperature (37 °C) and voltage = 0 V.

#### Ablated tissue

To determine the ablation zone (i.e., where cell death occurs), the Thermal Damage transformation model was included in the COMSOL model with a temperature threshold. The isothermal surfaces were plotted using the following parameters to determine the ablation zone: damage temperature 60 °C, damage time 1 s, necrosis temperature 100 °C, and enthalpy change 0 J/kg.

### In vitro experiments

We prepared a thermochromic tissue phantom (TCP) for direct visualization of ablation zone geometry and compared it with the FEM-simulated ablated tissue 3D geometries (Fig. 4A). The TCP tissue approximates the electrical properties of real tissue and provides a good in vitro setup for testing the bipolar electrodes. The TCP tissue was prepared using polyacrylamide gel with a thermochromic ink additive that permanently changes color from white to magenta when heated over 60 °C. The TCP was formulated as described by Mikhail et al. [26] and contained: deionized water 76.1 (v/v), 40% acrylamide/bis-acrylamide 17.5 (v/v), magenta MB60°C concentrate 5.0 (v/v) sodium chloride (NaCl) 0.9 (w/v), ammonium persulfate (APS) 0.14 (w/v), and N, N, N′, N′-tetramethylethylenediamine (TEMED) 0.14 (v/v). The electrical conductivity was adjusted to match the connective tissue since this is the most relevant parameter for the thermal Joule heating effect.

**Fig. 2:**
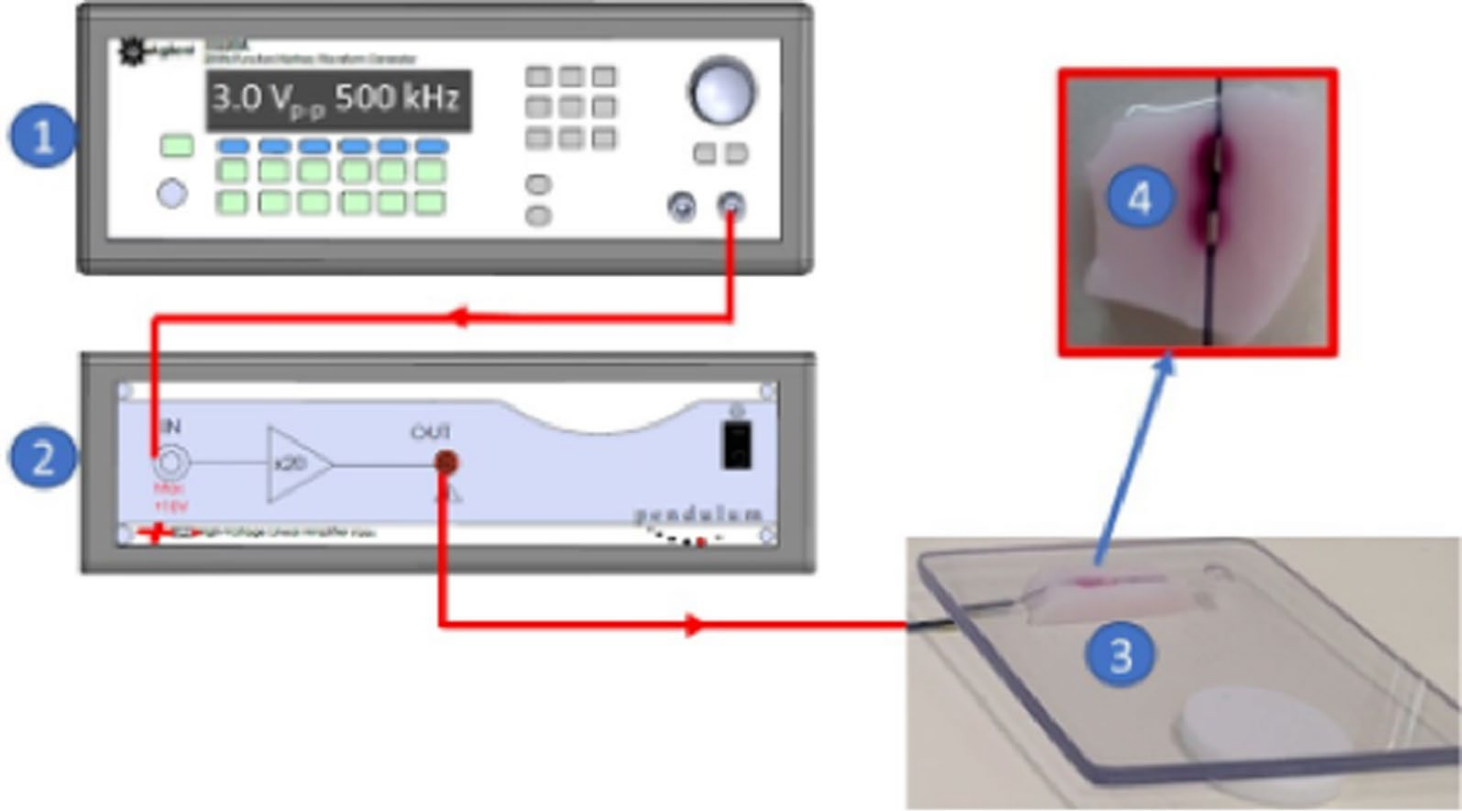
Thermochromic tissue phantom (TCP) ablation experimental setup. (1) Signal generator (3 Vp-p, 500 kHz) was amplified by a (2) 20 gain linear power amplifier (60 Vp-p, 500 kHz) and (3) applied to TCP with a bipolar electrode and an insulating plate on top of the dielectric-coated electrodes to maintain its position. (4) Top view of the TCP ablated by the bipolar electrodes in 60 s. Note, the electrodes are covered with translucent dielectric material as modeled in silico in Fig. 1B. Oscilloscope and precision meter not shown for clarity

The TCP was ablated with a bipolar electrode modeled in Fig. 1B and connected to a waveform generator and linear amplifier, as shown in Fig. 2. The bipolar electrode inside an artery as per the FEM in silico model was approximated with the TCP tissue resting flat and the bipolar electrode on top, as shown in Fig. 2. This experimental strategy was chosen to provide a common experiment framework for application to future ex vivo modeling on a dissected and split renal artery with the bipolar electrode placed on top. The exposed surface of the electrodes was in contact with the TCP and impregnated with saline solution, which mimics the conductivity of blood. An ablation signal of 3.0 V_p-p_ and 500 kHz was generated with an Agilent 33220A waveform generator (Agilent, Santa Clara, CA, USA) and amplified to 60 V_p-p_ with a Pendulum F20A fixed-gain linear amplifier (Pendulum, Stockholm, Sweden). A Tektronix TDS2001C oscilloscope (Tektronix, Beaverton, OR, USA) and a Fluke 8846A Precision Multimeter (Fortive, Everett, WA, USA) were connected to monitor and confirm that the appropriate ablation signal was applied to the TCP through the electrodes (not shown in Fig. 2 for simplification).

Ten in vitro experiments were conducted with the same setup as in Fig. 2. The 3D effects of the RF ablation on the TCP tissue were assessed by measuring the length and width with a ruler, with subsequent transection of the specimen to measure the height. The ablated area of TCP tissue was also quantified using ImageJ [29] to analyze the ten images of the TCP ablations scaled to 86 pixels = 1 mm using the Threshold Color tool with the default thresholding method (red color) and the following settings: hue = 217, saturation = 121, brightness = 68, color space HSB, and no dark background.

## Results

### RF ablation signal power

Table 2 summarizes the peak power delivered during the 60-s RF ablation signal application. The correlation between the FEM in silico model and in vitro TCP tissue ablation peak power was within 5%.

**Table 2:** RF ablation signal power

RF ablation experiment	Voltage^a^ (V)	Voltage^b^ (V)	Peak current (I)	Peak power (P)
	(V_p-p_)	(V_RMS_)	(mA)	(W)^c^
FEM Model	60	21.21	112	2.38
TCP Tissue	60	21.21	118	2.50

^a,b^Voltage applied to the tissue

^c^Power = V_RMS_ * I

### RF ablation catheter design

**Fig. 3:**
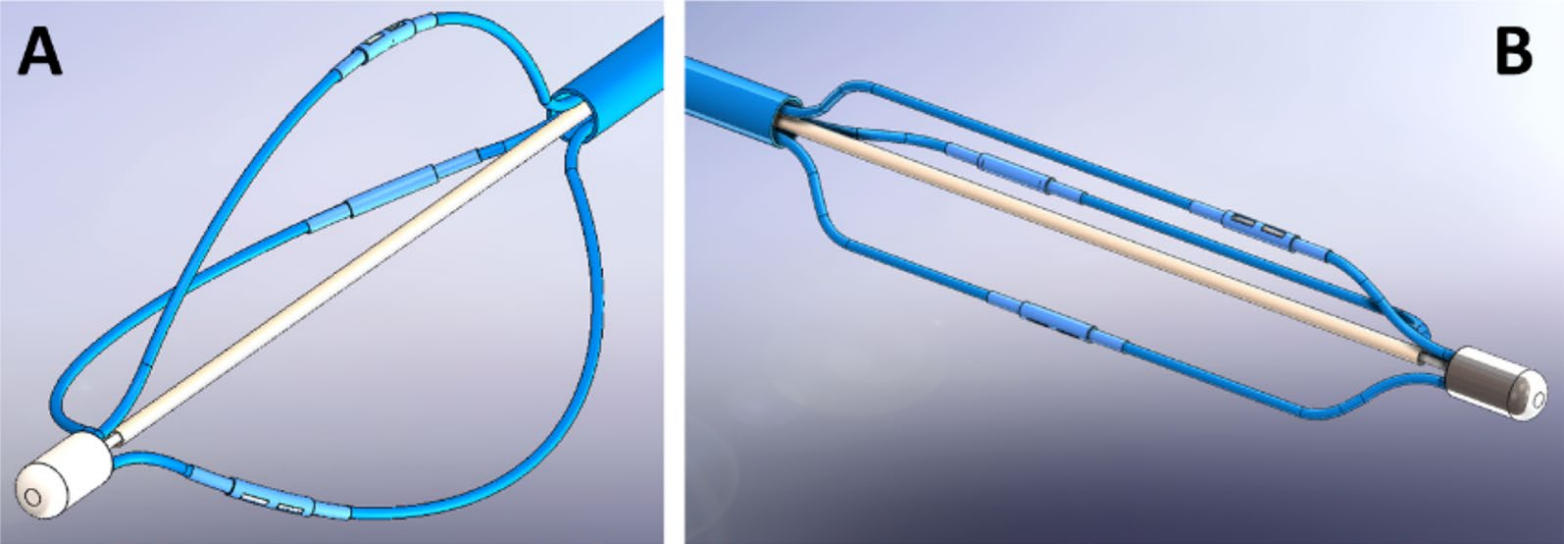
Graphical representation of the basket catheter with three splines and two electrodes per spline. **A** Basket deployed in an unrestrained open space showing Nitinol pre-shaped form covered by blue Pebax®. **B** Basket deployed inside a constraint space, such as an artery. Note the electrodes are not in the same circumferential plane to prevent renal artery stenosis after ablation

The bipolar electrode design in this study (Fig. 1) can be assembled in a 6 Fr catheter with four splines carrying either 2 or 4 electrodes per spline. Each spline is made of thermoplastic (Pebax®) extrusions with their inner lumen containing a pre-shaped nickel-titanium (Nitinol) and electrical wires (Fig. 3). The Nitinol provides a collapsible basket to be delivered through femoral access to the renal artery. It expands to the pre-shaped form creating firm connectivity between the electrodes and the artery inner walls. Each bipolar electrode can be independently selected for ablation of each quadrant in contact with the splines inside the artery (patent pending).

### In silico and in vitro comparisons

**Fig. 4:**
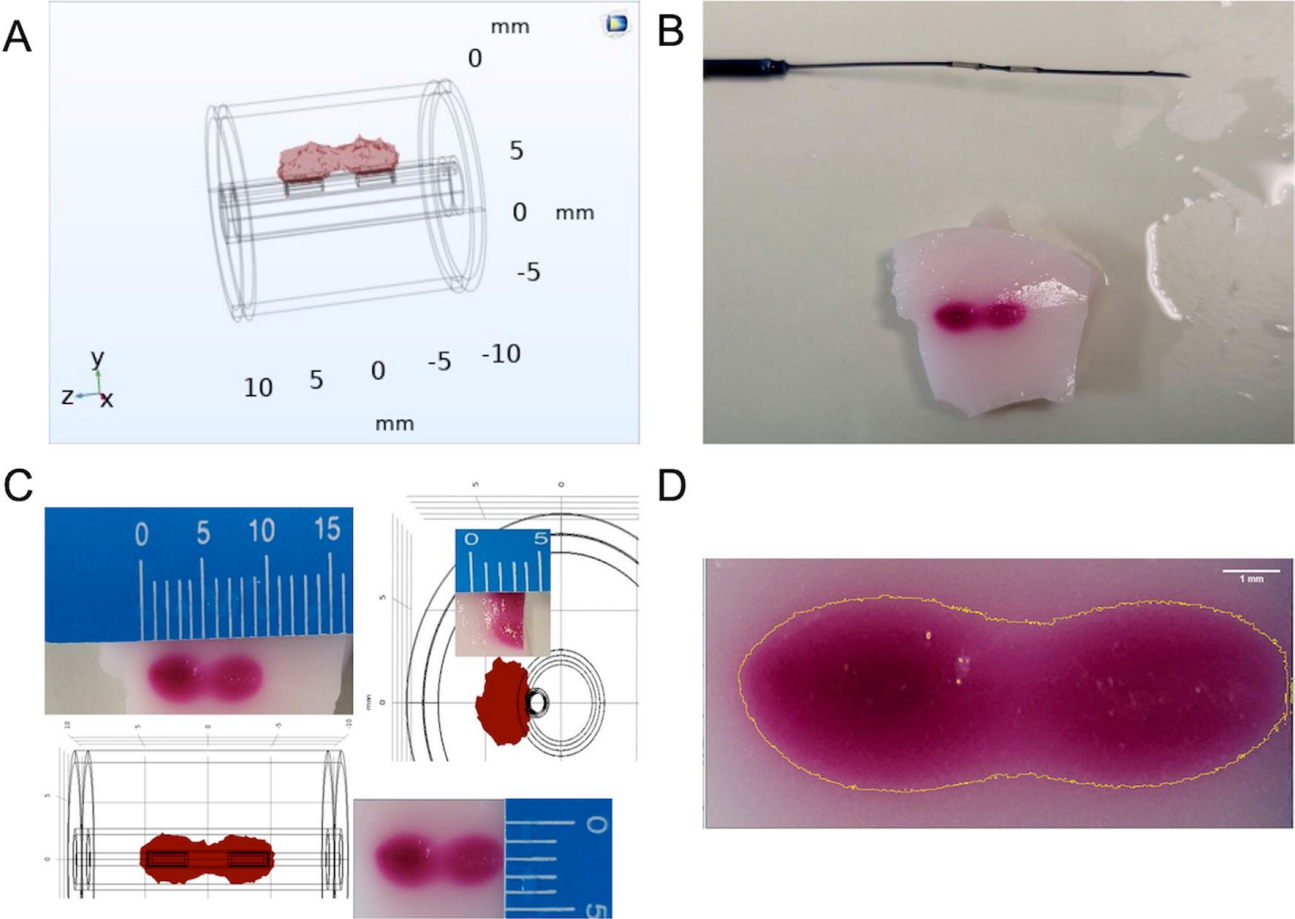
**A** Computer simulation of the isothermal ablation zone for a 500 kHz input signal with an amplitude of 60 Vp-p at 60 s of ablation. The computer model predicted an ablation zone of 4 mm × 10 mm × 4 mm (length × width × height) over the bipolar electrodes. **B** Thermochromic tissue phantom (TCP) after an ablation signal (500 kHz, 60 Vp-p) was applied for 60 s. The dark pink color change corresponds to the area where the temperature exceeded 60 °C. Note the similarities with the computer model in **A**. **C** Ablation zone comparison between computer model (**A**) and TCP tissue experiment (**B**). The ablation zone was 3 mm × 8.5 mm × 3.5 mm. Note the very close correlation between the experimental and simulated ablation volumes. **D** ImageJ processing of **C** illustrating the thresholding and measurement of the ablated zone

The isothermal plot from the computer model (Fig. 4A) predicted the tissue volume that reached an ablation temperature of ≥ 60 °C after 60 s of ablation due to the induced Joule heating. The ablated area consisted of two elongated lobes with an approximate maximum dimension of 4 mm × 10 mm × 4 mm (length × width × height).

The ablated TCP tissue (Fig. 4B) was measured and compared with the isothermal plot from the computer model (Fig. 4A) and is shown in Fig. 4C. Note the excellent geometrical correlation between simulated and experimentally ablated zones. For more accurate quantification, the images of ablated area of TCP tissue were quantified using ImageJ (Fig. 4D). The experimentally ablated zone measured 3.2 mm × 9.6 mm × 3.4 mm with a standard deviation of 0.12 mm for the ten experimental replicates. These values closely matched the model prediction of 4 mm × 10 mm × 4 mm, indicating that the computer model accurately predicted the RF ablation zone in vitro.

### Renal artery blood flow effects

**Fig. 5:**
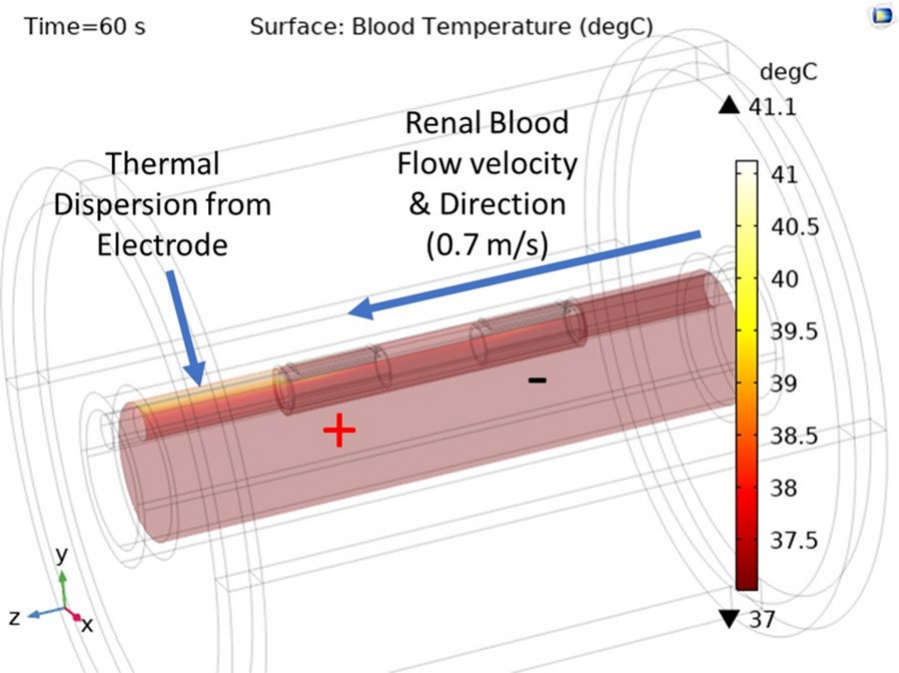
In silico FEM prediction of blood temperature after 60 s of RF ablation inside the renal artery flowing from the negative to the positive electrode. The blood temperature is predicted to be unaltered from the average body temperature of 37 °C, while thermal dispersion is predicted from the positive electrode and in the direction of the blood flow, which raises the arterial wall temperature to 41.1 °C

The RF ablation zones in Fig. 4 considered renal blood flow thermal dynamics. The blood temperature inside the artery was maintained at 37 °C, as shown in Fig. 5. Thermal dispersion from the positive electrode over the inside artery wall was noted in the direction of renal blood flow set at 0.7 m/s, a density of 1050 kg/m^3^, and normal blood pressure of 120/80 mmHg [27].

## Discussion

Resistant hypertension remains common and a clinical management challenge [1]. After initially disappointing clinical trial results, the technique's refinement has now resulted in the inconsistent demonstration of RD efficacy for patients with treatment-resistant hypertension [11, 12]. Additionally, the Food and Drug Administration (FDA) has not approved any device for RD except for investigational purposes, despite the many ongoing clinical trials for RD and suggesting a need for further catheter refinement.

Several RF ablation catheter devices with the CE mark are authorized for use in the European Union (EU) (Table 3). The configuration column states the operating mode between unipolar (requiring a ground or indifferent pad) and bipolar (no need for another external pad). All devices listed are unipolar devices except for the Vessix™ (Boston Scientific, Marlborough, MA) and our device, the only bipolar device. The downside of VessixTM is the inclusion of a balloon with bipolar electrodes [18]. This has the consequent disadvantage that once inflated, the balloon blocks renal blood flow during ablation. Our electrode design based on a basket catheter does not require a balloon, so it has the advantage of no renal blockage during ablation. Furthermore, there is no need for cooling, and the catheter has lower ablation times and power requirements than most existing designs.

**Table 3:** A comparison of CE-marked renal denervation catheters and our catheter. Adapted from [18]

Catheter	Energy	Configuration	Electrode	Balloon	Cooling	Delivery	Ablation time (s/artery)	Max. power (W)	Vascular access (Fr)
Simplicity™ (Medtronic)	RF	Unipolar	Single	No	Blood	Deflectable tip	540	8	6
Spyral™ (Medtronic)	RF	Unipolar	Multiple	No	Blood	Monorail	60	8	6
Vessix™ (Boston Scientific)	RF	Bipolar	Multiple	Yes	None	Over-the-wire	30	1	8
EnligHTN™ (St Jude)	RF	Unipolar	Multiple	No	Blood	Delectable tip	90	6	8
Iberis™^−^ (Terumo)	RF	Unipolar	Single	No	Blood	Delectable tip	540	8	4
Paradise™ (Recor)	US	Unipolar	Single	Yes	Close irrigated	Over-the-wire	50–150	30	6
Our design	RF	Bipolar	Multiple	No	Not required	Guided sheet	60	< 3	6

RF ablation devices have evolved from single unipolar electrodes (the original Symplicity HT1™, Medtronic, Santa Clara, CA, USA) to multi-electrode unipolar systems (Spyral™) [28] Here we designed a bipolar electrode for RF ablation with the electrode design, dimensions, geometry, and ablation area simulated and optimized via FEM in silico. Several software packages are available at varying costs based on the complexity and variables in the model. However, their benefit includes reducing further costs for design iterations that are simple to change in the FEM and predicting the results before designing and building the device and implementing in vitro experiments. The FEM simulation included the surrounding artery and connective tissue's electrical properties to predict the ablation zone dimensions to achieve renal nerve ablation. A TCP tissue was used to compare the in vitro ablation zone with FEM in silico* simulations*. A limitation of the TCP is its formulation with only one set of electrical characteristics; consequently, the experimental strategy was to define the electrical properties of the renal artery walls in direct contact with the surface of the electrodes to provide conservative experimental data on the voltage/ablation time required for further optimization when moving to ex vivo experimentation. Another limitation of the TCP is measurements of ablation zones. TCP starts changing color at 60 °C, and there is considerable visual variability to determine the exact location where the TCP starts to display temperature color transition. Nevertheless, the TCP allows visual and quantitative comparison of the geometry, shape, and form of ablation, which very closely matched the computational simulation. Therefore, this model will be helpful for further optimizing catheter design for accurate renal nerve ablation in animal models in vivo to pave the way for clinical validation.

This computational model of RF ablation can be used to estimate ablation zones in the renal artery for RD in patients with hypertension. The model provides an efficient iterative platform to design and refine RF ablation electrodes for targeted ablation geometries and dimensions. The model can be extended to other anatomical landmarks to predict the RF ablation pattern in the targeted zone. This model is complemented by the TCP to provide a relatively simple and cost-effective in vitro medium for quick feedback to the computational model from RF ablation experiments, allowing short iteration times during catheter development compared to traditional, costly, and time-consuming animal and human trials.

## Conclusions

Our bipolar RF ablation electrode deserves further study and development as a potential RD device. The bipolar configuration and innovative electrode design ensure that the current density is focused on the target tissue, reducing the energy transferred to renal blood, reducing the need for cooling. Furthermore, the electrode window can be modified in shape and dimensions based on the intended location and geometry desired for ablation. This model and TCP can be used to investigate other ablation applications, e.g., tumor ablation, to design an electrode for the specifically intended zone. Further ex vivo and in vivo studies are warranted to further validate this new bipolar catheter system.

## Data Availability

All data generated or analyzed during this study are included in this published article.

## Abbreviations

AHA: American Heart Association
FDA: Food and drug administration
FEM: Finite element modeling
RD: Renal denervation
RF: Radiofrequency
RH: Resistant hypertension
TCP: Thermochromic tissue phantom
HT: Hypertension
